# Co-culture of bone marrow-derived macrophages with aged primary myoblasts

**DOI:** 10.1093/biomethods/bpag041

**Published:** 2026-07-11

**Authors:** Ryan Arsenault, Anthony Ziadeh, Alexander Green, Junio Dort

**Affiliations:** School of Pharmaceutical Sciences, Faculty of Medicine, University of Ottawa, Ottawa, ON K1H 8M5, Canada; Department of Biochemistry, Microbiology and Immunology, Faculty of Medicine, University of Ottawa, Ottawa, ON K1H 8M5, Canada; Interdisciplinary School of Health Sciences, Faculty of Health Sciences, University of Ottawa, Ottawa, ON K1N 6N5, Canada; Department of Laboratory and Clinical Sciences, School of Health Sciences, Algonquin College of Applied Arts and Technology, Ottawa, ON K2G 1V8, Canada; School of Pharmaceutical Sciences, Faculty of Medicine, University of Ottawa, Ottawa, ON K1H 8M5, Canada; Department of Biochemistry, Microbiology and Immunology, Faculty of Medicine, University of Ottawa, Ottawa, ON K1H 8M5, Canada; Department of Cellular and Molecular Medicine, Faculty of Medicine, University of Ottawa, Ottawa, ON K1H 8M5, Canada

**Keywords:** skeletal muscle, cell isolation, co-culture, macrophages, myoblasts, aging

## Abstract

Skeletal muscle regeneration depends on the coordinated activity of multiple cell populations within the muscle stem cell niche, most prominently macrophages, which undergo dynamic phenotypic transitions essential to tissue repair. Aging disrupts this process, impairing macrophage signaling and muscle stem cell function in ways that are not yet fully understood. Existing approaches (e.g. conditioned media transfer and indirect transwell co-culture) fail to recapitulate the direct cell-to-cell contact required for continuous, reciprocal regulation throughout the regenerative cascade. Here, we present a protocol for the isolation of bone marrow-derived and tissue-resident macrophages from skeletal muscle, together with a direct co-culture methodology designed to interrogate macrophage–myoblast interactions in an age-relevant context.

## Introduction

Muscle injury triggers an acute and highly coordinated inflammatory response, characterized by a sequential recruitment of immune cells, most prominently macrophages, that play essential roles in tissue repair [1, 2]. Upon injury, circulating monocytes rapidly invade the damaged site, where they differentiate into macrophages to orchestrate tissue repair through sequential functional transitions [3]. While macrophages are commonly categorized as pro-inflammatory (M1) or anti-inflammatory (M2), transcriptomic studies have demonstrated that their activation exists along a continuum of functional states, with Ly6C^hi^ and Ly6C^lo^ populations only partially overlapping with classical M1 and M2 gene expression profiles [2]. Under the traditional framework, classically activated M1 macrophages arise in response to pro-inflammatory signals, including damage-associated molecular patterns (DAMPs) released from damaged tissue, microbial products such as bacterial lipopolysaccharide (LPS), and cytokines such as interferon- γ (IFN-γ), thereby acquiring a pro-inflammatory phenotype [2, 4–8]. Key surface markers of this phenotype include CD80, CD86, TLR2, and TLR4 [9–12]. Conversely, the alternatively activated, anti-inflammatory M2-like macrophages are polarized by factors such as interleukin (IL)-4 and IL-13, and are characterized by high expression of CD206, CD163, ARG1, and CHIL3 [4, 13–15].

Within this spectrum, macrophages exert multifaceted control over skeletal muscle regeneration by coordinating inflammation, myogenesis, fibrosis, and revascularization [2]. Following injury, early-stage proinflammatory macrophages clear necrotic debris via phagocytosis, a strict prerequisite for effective muscle regeneration, while simultaneously releasing factors (e.g. prostaglandin E2) to stimulate myogenic cell expansion [2, 16–18]. These macrophages also regulate the accumulation of fibroadipogenic progenitors via tumor necrosis factor α (TNF-α)-induced apoptosis, preventing excessive fibrofatty deposition [2]. As inflammation resolves, macrophages transition to an anti-inflammatory phenotype, releasing various factors (e.g. IL-4/10, IGF-1) that drive myoblast differentiation, fusion, and myofiber growth/regeneration [2]. More recently, macrophage metabolism has emerged not only as a determinant of macrophage function but also as a source of paracrine signals that shape the regenerative niche [19]. In particular, macrophage-derived metabolites, including glutamine, have been shown to directly regulate myogenic progenitor cell function and accelerate muscle regeneration, highlighting metabolite-mediated communication as an additional mechanism by which macrophages coordinate skeletal muscle repair [20–22].

This spatiotemporal coordination is vital; experimental models featuring macrophage depletion consistently result in persistent necrotic tissue, impaired myogenesis, and defective muscle regeneration [16, 17, 23]. Aging profoundly perturbs the finely tuned macrophage response required for muscle regeneration, driving the age-related decline in regenerative capacity [24, 25]. In aged muscle, macrophages exhibit altered phenotypes and impaired paracrine signaling, reducing their ability to support myoblast expansion [7, 26–29]. Investigating these complex, age-sensitive interactions requires advanced *in vitro* models capable of capturing the direct cellular crosstalk between macrophages and myoblasts.

Direct co-culture models have had a great impact on research in recent years. For instance, co-culture models have already been developed to study the interaction of skin cells and immune cells [30]. Co-cultures mimicking neuromuscular circuits have also been studied in the context of diseases such as amyotrophic lateral sclerosis [31]. However, no direct co-culture of macrophage-differentiating myoblasts has been reported in the context of aging. The platform outlined here addresses this methodological gap via multiple distinct advantages. First, it enables the interrogation of direct macrophage-myoblast contact, overcoming a key limitation of previously described indirect transwell models [32, 33]. Maintenance of this physical contact is required throughout all stages of muscle regeneration to enable continuous, reciprocal regulation of both cell types [34, 35]. Second, this approach preserves the physiological paracrine signaling, while avoiding the concentration-dependent toxicity and stability artifacts associated with conditioned media transfer protocols. Another key advantage of our approach is that macrophages are polarized before co-culture, ensuring a defined and consistent phenotype before interacting with myoblasts. In particular, it enables a more precise assessment of how modulation of macrophage behavior by therapeutic candidates subsequently influences myoblast responses. Finally, compared to costly, lengthy, and complex *in vivo* models, this protocol provides a rapid platform to investigate macrophage-myoblast interactions and their dysregulation in advanced age and pathological conditions.

## Materials and methods

### Generation of bone marrow-derived macrophages

*Note:* Here, we used >20-month-old male C57BL/6 mice to establish this protocol.

Equipment:

1. Refrigerated centrifuge
2. Sterile surgical kit (including scissors and forceps)
3. Hemocytometer or automated cell counter

Materials:

1. DMEM 1X with 4.5 g/L Glucose & L-Glutamine without Sodium Pyruvate (Wisent; 319-015-CL)
2. Antibiotic/Antimycotic (Gibco; 15240-062)
3. Heat-inactivated fetal bovine serum (FBS) (Corning; 35-087-CV)
4. Mouse Macrophage colony-stimulating factor (M-CSF) (Miltenyi Biotech; 130-094-129)
5. Bacterial LPS (Millipore; L4391-1MG)
6. IFN-γ (Millipore; 11276905001)
7. Red blood cell (RBC) lysis buffer (Miltenyi Biotech; 130-094-183)
8. 0.22 μm filter unit (Millipore; SLGP033RS)
9. 70% Ethanol
10. Sterile PBS
11. Sterile Petri dish (one per mouse)
12. 0.5 ml microcentrifuge tubes (two per mouse)
13. 1.5 ml microcentrifuge tubes (two per mouse)
14. 10 ml syringe (one per mouse)
15. 25-gauge needle (one per mouse)
16. Sterile 70 µm filter (one per mouse)
17. 50 ml tubes (one per mouse)
18. 15 ml tubes (one per mouse)
19. 12-well plate (one per mouse)

To be prepared before the start of protocol:

1. Wash buffer: PBS with 2% FBS, filter through a 0.22 μm filter unit. Fifty milliliters per mouse is recommended throughout the protocol. Keep wash buffer on ice.
2. Differentiation media: DMEM (4.5 g/L glucose) media with 10% FBS, 1% antibiotic/antimycotic. Prepare media with fresh M-CSF (10ng/ml) before each use. Pre-warm at 37°C.
3. Pre-chill centrifuge to 4°C.

**Figure 1:**
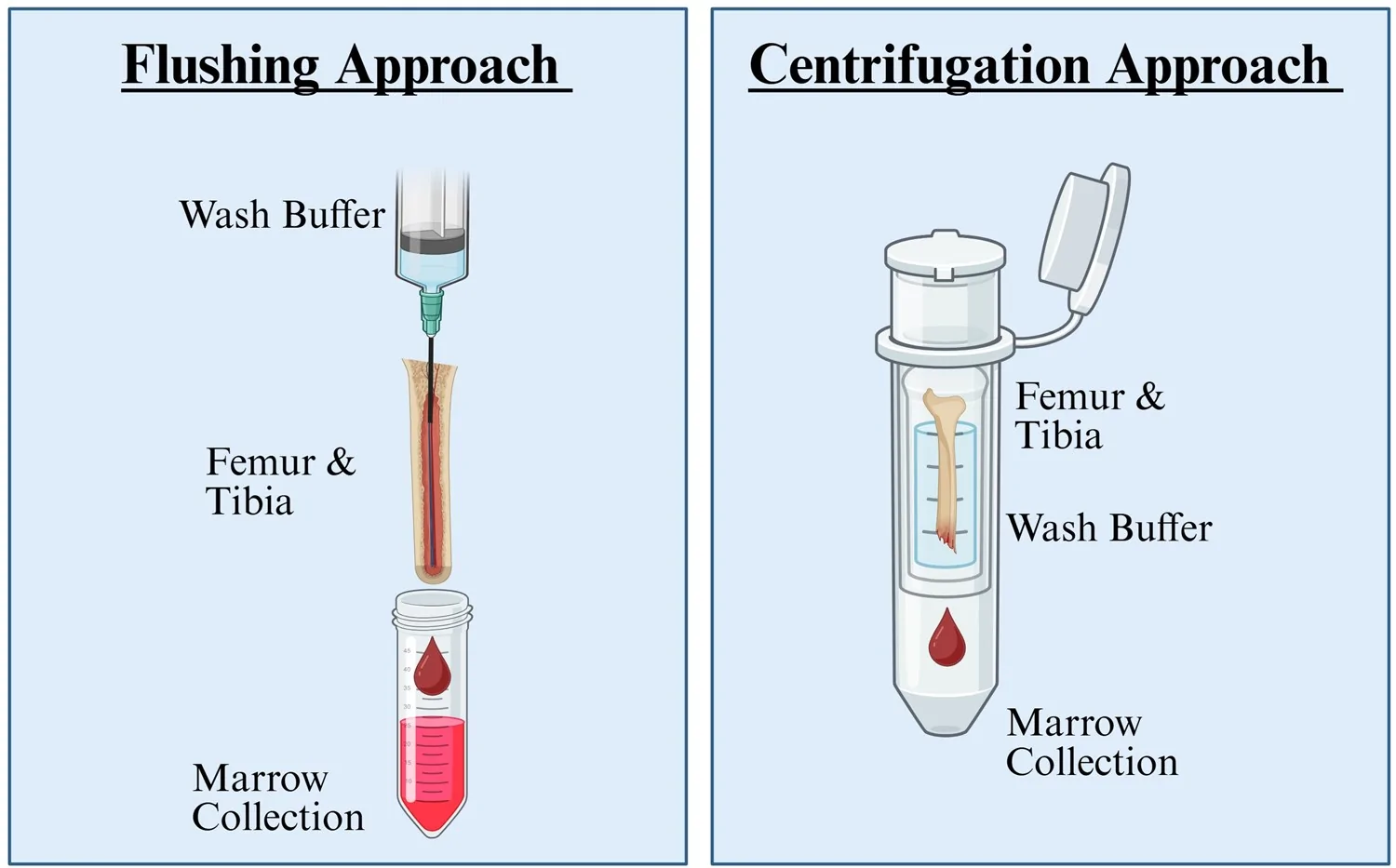
Schematic representation of the flushing and centrifugation approaches.

**Figure 2:**
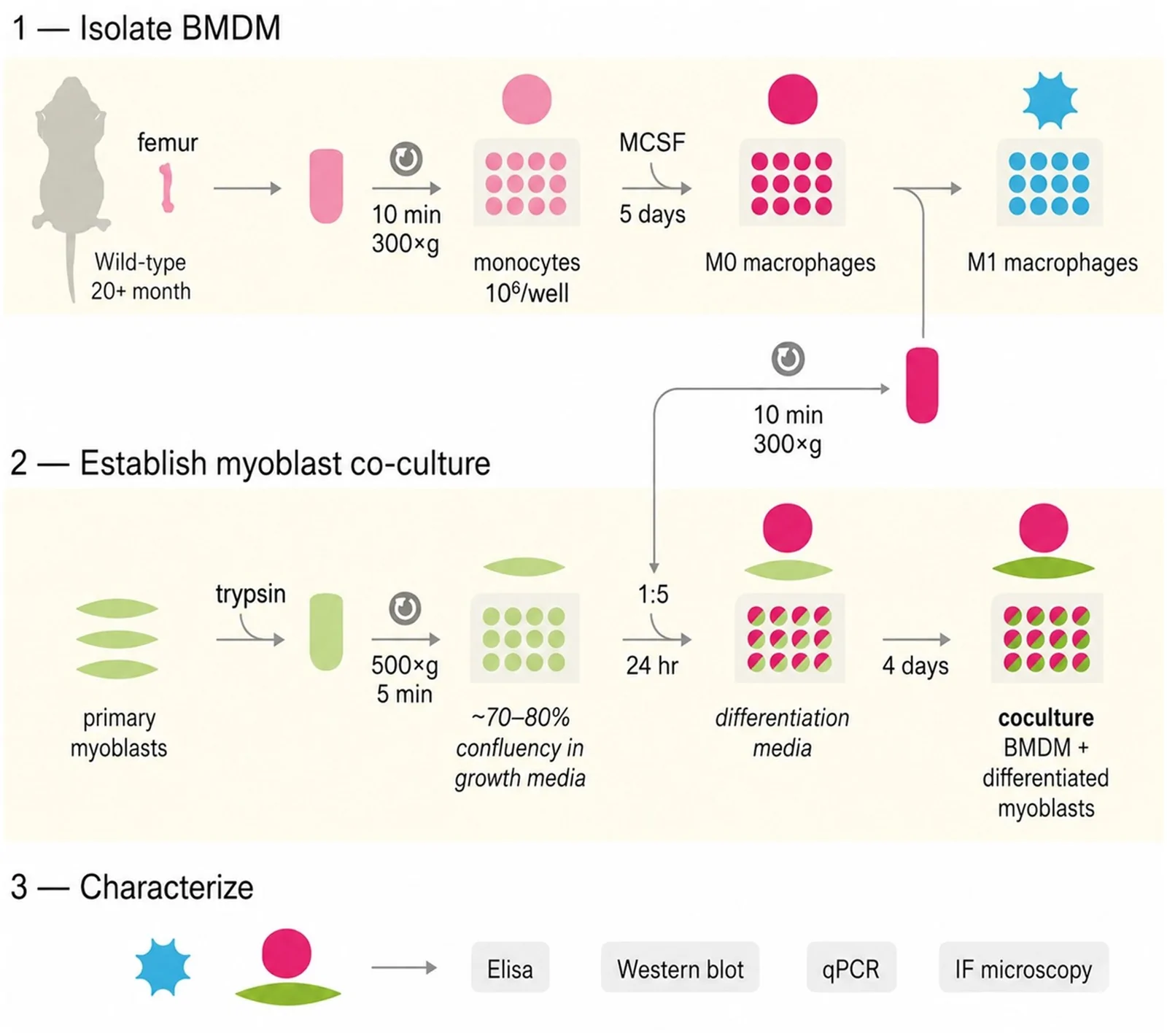
Protocol overview. (1) Hindlimbs were collected from aged mice, and monocytes were isolated from the bone marrow and cultured in growth medium supplemented with macrophage colony-stimulating factor (M-CSF) for 5 days to generate M0 macrophages. Macrophages were then stimulated (M1) with bacterial lipopolysaccharide (LPS) and interferon-γ (IFN-γ) for 24–48 hours. Macrophages can be collected at M0 and M1 stages for phenotypic characterization (e.g. immunofluorescence or Western blot of key markers: CD206/CD163) and/or downstream co-culture experiments. (2) Primary myoblasts isolated from aged mice were plated in collagen-coated 12-well plates and allowed to adhere for 24 hours. Macrophages were then directly added at a 1:5 macrophage-to-myoblast ratio, and cells were differentiated for 4 days in low-serum culture medium until myotubes were formed. (3) Following differentiation/fusion, samples were either fixed for microscopy or collected for downstream molecular and cellular analyses.

**Figure 3:**
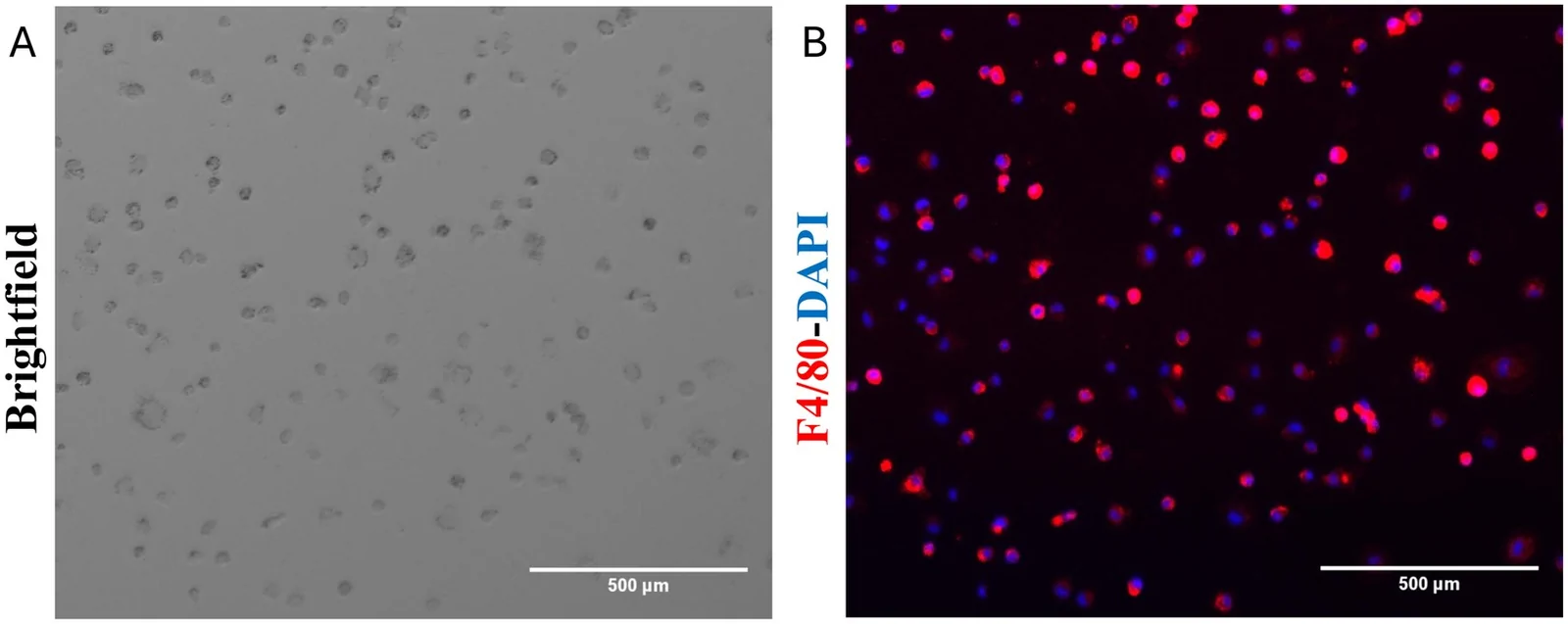
Representative images of differentiated bone marrow-derived macrophages. (**A**) Brightfield and corresponding (**B**) immunofluorescence images of macrophages stained for F4/80 (Bio-Rad; MCA497R) and DAPI (Invitrogen; H3570).

Protocol:

1. Anesthetize the mouse, shave the hindlimbs and clean with 70% ethanol. Euthanize the mouse via cervical dislocation and quickly proceed with step 2. *ATTENTION*: The methods of euthanasia may vary. Always follow your institutional guidelines in accordance with your approved experimental animal protocol.
2. Using scissors, snip around the ankle, then toward the inner thigh and peel the skin back to fully expose the leg muscles. Clean the femur and tibia by removing the majority of the surrounding muscles.
3. Harvest the femur and tibia by cutting near the hip.
*Note: If the femur is not fully collected, this will significantly decrease the cell yield.*
4. Holding the limb by the foot above an ice-cold tube filled with 10 ml wash buffer, cut off the foot, allowing the bones to fall into the tube.
5. Repeat steps 2–4 with the other limb.
6. Pour the buffer containing the cleaned bones into a small Petri dish and remove any obstructive muscle.
7. Carefully separate the femur from the tibia by splitting the knee. Carefully cut off the epiphyses of each bone; be cautious, as the bones are easily broken.
*Note: Only remove enough to fully insert the needle into the medulla.*
8. Collect the bone marrow either by flushing or centrifugation (Fig. 1). Note that poor bone marrow collection will significantly reduce monocyte yield.
*Flushing approach:* flush each bone marrow cavity with cold wash buffer into a 50 ml Falcon tube using a fine needle and syringe. It is recommended that each bone be flushed with a minimum volume of 10 ml to ensure complete marrow collection. Well-flushed bones will appear transparent.
*Centrifugation approach:* alternatively, after cutting open the bones at the epiphyses, place them, cut sides down, into a 0.5 ml microcentrifuge tube with a pierced bottom (which can be done using a 25G needle), nested inside a 1.5 ml tube. Add 100–150 µL of cold wash buffer and centrifuge at 4000 × *g* for 15 s at 4°C. Transfer the pelleted marrow to a 50 ml Falcon tube.
9. Repeat steps 7 and 8 with the other limb.
10. Homogenize by gently pipetting up and down using a P1000 until no large clumps of marrow remain, and filter through a 70 µm filter into a new 50 ml tube on ice. Incomplete homogenization will significantly reduce the final monocyte yield.
11. Discard filter and centrifuge at 300 ×g for 10 minutes at 4°C. While centrifuging, prepare 1X RBC lysis buffer according to the manufacturer’s instructions.
12. Discard supernatant and resuspend pellet in 5 ml of RBC lysis buffer, mixing gently by pipetting. Incubate at room temperature for 10 minutes. After incubation, add 10 mL of cold wash buffer, mix gently and centrifuge at 300 × g for 10 minutes at 4°C.
13. Resuspend the cell pellet in pre-warmed bone marrow-derived macrophage (BMDM) differentiation medium (supplemented with 10 ng/ml M-CSF), determine the cell concentration using a hemocytometer or an automated cell counter, and adjust the cell suspension to ∼5 × 10^5^ cells/ml before seeding.
14. Plate cells at a high density (∼1–1.5 ml per well in a 12-well plate) and place directly into the incubator at 37°C and 5% CO_2_. Cells will adhere quickly, within 2–3 hours. Change media after 24 hours.
15. Culture BMDMs for at least 4 and up to 6 days, replacing medium every 24 hours with fresh differentiation medium containing 10 ng/ml M-CSF (Fig. 2).
16. To verify macrophage enrichment, differentiated BMDMs may be characterized by flow cytometry using established macrophage markers such as F4/80 and CD11b [36, 37]. Under the M-CSF differentiation conditions described here, previous studies have reported macrophage enrichment exceeding 90% [36–38]. Using F4/80 as a pan-macrophage marker, we consistently observed uniform positive staining across the differentiated cell population, indicating a highly enriched macrophage culture (Fig. 3). However, we recommend flow cytometric assessment when quantitative evaluation of culture purity is required for downstream applications.
17. Following complete differentiation, macrophages can be M1 polarized using 100 ng/ml LPS and 25 ng/ml IFN-γ for 24–48 hours. Depending on the experimental objective, the test molecule can be added either during M1 polarization (to assess potential inhibition of phenotype acquisition) or after polarization (to evaluate effects on already polarized macrophages).
18. Differentiated macrophages can then be used in downstream applications, including, but not limited to: RNA-sequencing, ELISA, qPCR, western blot, immunofluorescence and co-culture experiments (Fig. 2).

### Alternative isolation of tissue-resident macrophages

**Figure 4:**
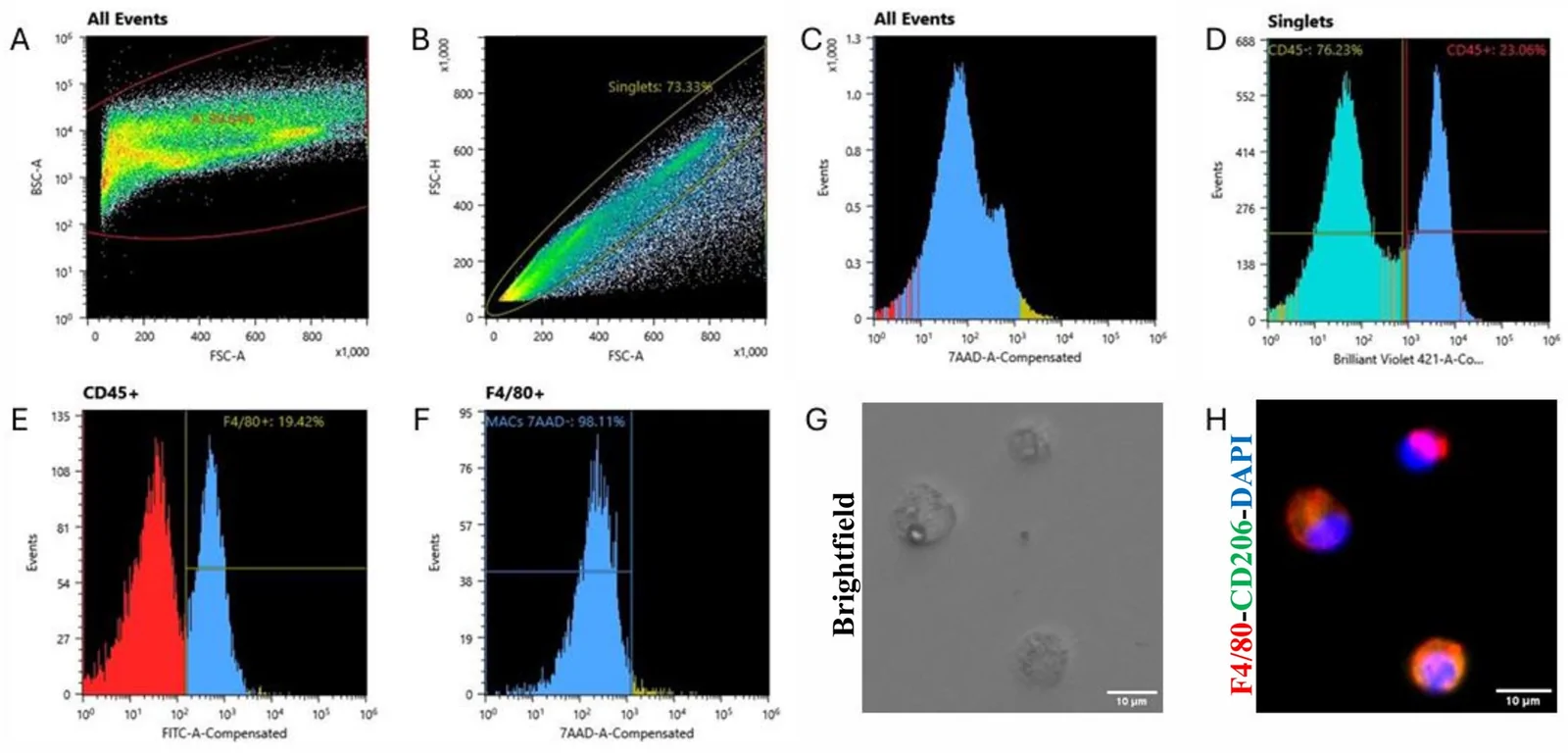
Gating strategy to isolate skeletal muscle-resident macrophages from >20-month-old mice. (**A**–**F**) CD45^+^cells are gated from singlet events, and then F4/80^+^ cells are gated from CD45^+^ events. Finally, F4/80^+^ and 7AAD^-^ events are gated to isolate live F4/80^+^ cells. Representative (**G**) brightfield and (**H**) immunofluorescence images of FACS-sorted macrophages stained with the pan-macrophage marker F4/80, anti-inflammatory CD206 and DAPI).

Although the direct co-culture system described in this manuscript was optimized using BMDMs, tissue-resident macrophages represent a physiologically relevant alternative for investigating macrophage–myogenic cell interactions [2, 39–41]. Accordingly, we adapted a previously published fluorescence-activated cell sorting (FACS) protocol [42] to isolate resident macrophages directly from the skeletal muscle of aged mice (Fig. 4). To reduce non-specific background staining commonly observed in aged tissues, we introduced an Fc receptor blocking step (BioLegend, Cat#156603) before primary antibody incubation (CD45/BV421, BioLegend, Cat#147719; F4/80/FITC, Miltenyi Biotech, Cat# 130-117-509, 1:1000). Using a Sony SH800 cell sorter, and the gating strategy shown in Fig. 4, resident macrophages were successfully isolated from digested skeletal muscle and maintained on collagen-coated plates in high-glucose DMEM supplemented with 20% FBS and 1% antibiotic/antimycotic. Although this isolation protocol has not yet been validated in the co-culture system described herein, it provides investigators with an alternative source of macrophages that can be incorporated into similar experimental paradigms depending on the biological question. It is important to note, however, that protocols relying on freshly isolated muscle-resident macrophages are often limited by lower cell yield, greater biological variability, and reduced experimental flexibility compared with BMDMs [41].

### Myoblast differentiation and co-culture with BMDMs

*Note:* Primary myoblasts should be isolated and expanded (passages 2–7) before initiating this protocol. These cells can be isolated using established enrichment methods, including gradual pre-plating, magnetic-activated cell sorting (MACS), or FACS [42–44]. For the experiments described here, primary myoblasts were isolated by MACS from skeletal muscles of >20-month-old male C57BL/6 mice.

Materials and equipment:

1. Ham’s F10 (Wisent; 318-050-CL)
2. DMEM 1X with 1.0 g/L Glucose, L-Glutamine & Sodium Pyruvate (Wisent; 319-010-CL)
3. Human FGF basic (Wisent; 511-126-QU)
4. Horse serum (HS) (Wisent; 065110)
5. Antibiotic/Antimycotic (Gibco; 15240-062)
6. Heat-inactivated FBS (Corning; 35-087-CV)
7. 0.05% Trypsin-EDTA (ThermoFisher; 25300054)
8. Aged primary myoblasts
9. Collagen stock (4.1 mg/ml; Corning, Cat. #354236)
10. Culture dishes
11. Cell counter or hemocytometer

To prepare before starting protocol:

1. Collagen solution for plate coating: Prepare sterile 0.02 N acetic acid by diluting 0.5 ml glacial acetic acid in 435 ml distilled water and filtering through a 0.2-µm filter. Dilute 1 ml of collagen stock (4.1 mg/ml; Corning, Cat. #354236) in 40 ml of sterile 0.02 N acetic acid before use.
2. Myoblast growth media: Hams F10, 20% FBS, 1% Antibiotic/Antimycotic, 12 ng/ml FGF.
3. Myoblast differentiation medium: 50% DMEM low glucose, 50% Ham’s F10, 5% HS, 1% antibiotic/antimycotic.
4. Pre-warm trypsin: Pre-warm trypsin to 37°C. Ensure trypsin is warmed well, as this will help significantly with detaching BMDMs from the culture dish.

Protocol:

*Note:* Myoblasts at high passage number (≥10) should not be used, as their ability to differentiate is drastically dysregulated.

**Figure 5:**
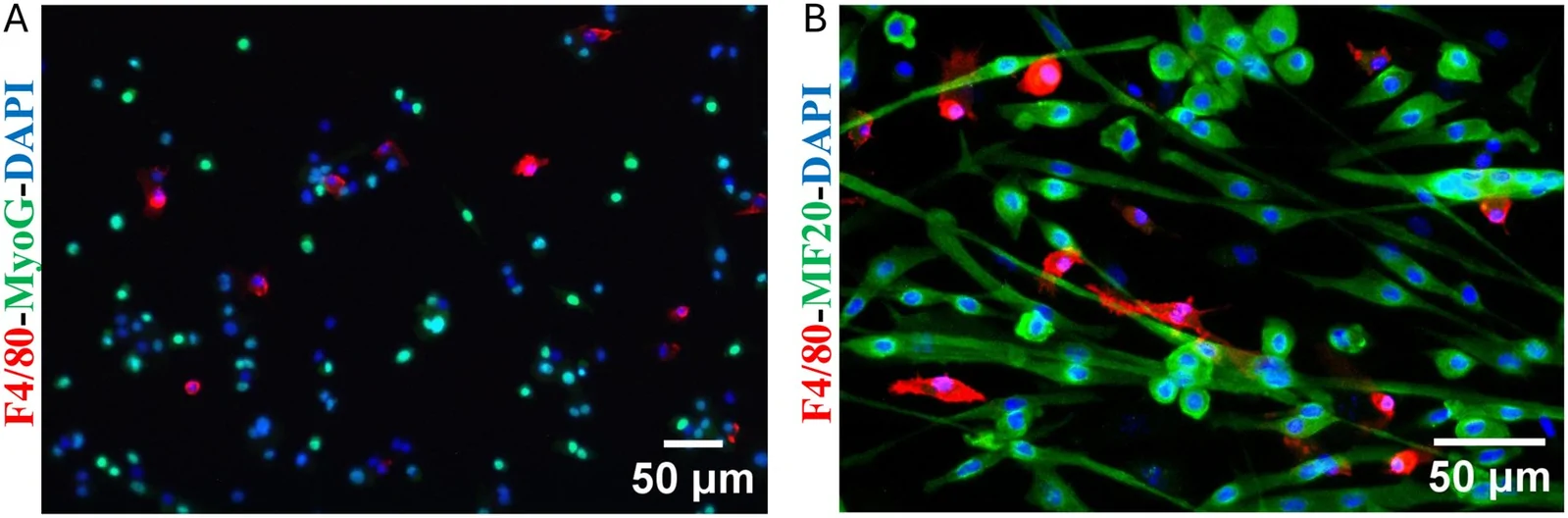
Representative images of the co-culture model. (**A**) Twenty-four-hour co-culture of bone marrow-derived macrophages (F4/80^+^DAPI) with primary myoblasts (myogenin, ab124800+DAPI). (**B**) Four days of macrophage (F4/80^+^DAPI) co-culture with primary myoblasts. Multi-nucleated myotubes are stained for myosin heavy chain (MyHC, MF20, DSHB + DAPI). All images were taken on the ThermoFisher EVOS M5000 fluorescent microscope.

Day 1:

1. Cover the entire surface of the culture dishes with the collagen solution, aspirate the excess, and allow the dishes to air-dry overnight in a sterile tissue culture hood. Alternatively, allow the coated dishes to air-dry under a sterile tissue culture hood with the lids removed for approximately 2.5 hours.
2. Plate primary myoblasts onto collagen-coated culture dishes and expand them in myoblast growth medium.
3. Collect myoblasts from the primary culture dishes using trypsin and centrifuge at 500 × *g* for 5 minutes at room temperature.
4. Discard the supernatant and resuspend the pellet in growth medium. Count the cells and plate them on a collagen-coated 12-well plate at a density of ∼90 000/cm^2^ (300 000 in a well/12 wells). The goal is to get 80%–85% cell confluency before starting the co-culture. Culture cells for at least 24 hours in an incubator set at 37°C and 5% CO_2._
  Note: We recommend determining the proliferation rate of the myoblasts beforehand (e.g. using an Incucyte or equivalent live-cell imaging system) to accurately estimate the appropriate seeding density for reaching 80% confluency when initiating co-culture with macrophages.
  Day 2:
5. Detach BMDMs with pre-warmed trypsin for 5 minutes at 37°C, gently tap the plate to detach any remaining adherent cells, neutralize with complete culture medium, collect the cells in a 15-ml tube, and centrifuge at 500 × *g* for 5 minutes at room temperature.
6. Count BMDMs and resuspend to a volume corresponding to a 1:5 macrophage-to-myoblast ratio per ml (∼60–80 000 BMDMs/ml). Aspirate myoblast media and add 1 ml of BMDM solution to each well. Swirl the plate very gently to ensure distribution of BMDMs, but be careful not to detach myoblasts.
7. Replace media every 24 hours, allowing myoblasts to differentiate. In approximately 4–5 days, myoblasts will differentiate and fuse into multinucleated myotubes (Fig. 5).
8. Myoblasts, myotubes and macrophages can be analyzed by immunofluorescence (Fig. 5).

## Results

Several standard morphological and differentiation metrics can be quantified from immunofluorescence images (e.g., Myogenin or MyHC staining combined with F4/80 staining, with DAPI nuclear staining).

### Differentiation index

The differentiation index reflects the proportion of myogenic cells that have initiated terminal differentiation.

Calculation:


$$\text{Differentiation}\;\text{index}\;(\%)=\frac{\text{Number}\;\text{of}\;\text{nuclei}\;\text{within}\;\text{MyHC-positive}\;\text{cells}}{\text{Total}\;\text{number}\;\text{of}\;\text{nuclei}}\times 100.$$

Nuclei count should exclude those from F4/80^+^ cells (macrophages).

### Fusion index

To assess myoblast fusion into multinucleated myotubes.

Calculation:


$$\text{Fusion}\;\text{index}\;(\%)=\frac{\text{Number}\;\text{of}\;\text{nuclei}\;\text{in}\;\text{myotubes}\;\text{containing}\geq 2\;\text{nuclei}}{\text{Total}\;\text{number}\;\text{of}\;\text{nuclei}}\times 100.$$

Nuclei count should exclude those from F4/80^+^ cells (macrophages).

### Myotube size (diameter)

For each myotube, the widest point along its length is identified visually, and a single perpendicular measurement should be taken across that region (in µm).

## Discussion

Here, we describe a robust protocol for the isolation and culture of murine BMDMs, their polarization, and direct co-culture with primary myoblasts. We demonstrate that these macrophages can be maintained in direct contact with aged myoblasts throughout differentiation into multinucleated myotubes, providing a physiologically relevant *in vitro* system to investigate how macrophages and their secreted factors regulate myogenic cell function in an age-relevant context.

This co-culture system provides a controlled experimental platform to distinguish direct macrophage-derived effects on myogenic cells from the broader complexity of the *in vivo* regenerative niche. Such an approach is particularly valuable considering emerging evidence that macrophages regulate skeletal muscle regeneration not only through cytokines and growth factors but also through metabolite-mediated signaling, including macrophage-derived glutamine [2, 19–22, 38]. This co-culture system complements *in vivo* studies, providing a precise platform to dissect how macrophage-derived metabolites and soluble mediators mechanistically regulate muscle regeneration. In addition, these methodologies can be adapted to investigate how distinct macrophage populations regulate inflammation, regeneration and therapeutic responses during aging and neuromuscular disease.

Although the BMDMs generated using this protocol provide a well-established and highly reproducible model for investigating macrophage–myoblast interactions, they do not fully recapitulate the phenotypic diversity and dynamic transitions of resident and monocyte-derived macrophage populations present during skeletal muscle regeneration. Because macrophage phenotype is continuously shaped by the local tissue microenvironment, findings obtained using this system should be interpreted within the context of this experimental model [2]. Nevertheless, its experimental simplicity and reproducibility make it a valuable platform for dissecting the molecular and cellular mechanisms underlying macrophage function. We have successfully applied this exact protocol to primary myoblasts and BMDMs isolated from young adult D2-mdx mice, demonstrating its applicability to younger animals and disease models. Depending on the specific age, strain, or disease context, minor optimization of parameters such as cell seeding density and culture duration may be considered.

## Data Availability

No new data were generated or analyzed in support of this study.
